# 

**Published:** 1884-12

**Authors:** 


### BOOKS AND PAMPHLETS RECEIVED.

*A Practical Treatise* on Disease in Children, By Eustace Smith, M. D., Fellow of the Royal College of Physicians; Physician to his Majesty the King of the Belgians; Physician to the East London Children's Hospital, and to the Victoria Park Hospital for Diseases of the Chest. New York: Wm. Wood & Co. 1884.

*Notes* on the Treatment of Trachoma by Jequirity. *A Clinical Study* of Syphilis of the Eye, and its Appendages. *Mumps as a Cause of Sudden Deafness.* Reprints from Medical Journals, By Leartus Connor, A. M., M. D., Ophthalmic Surgeon of Harpers Hospital, Detroit, Michigan.

*A Practical Treatise on the Diseases of the Ear,* including a sketch of Aural Anatomy and Physiology, By D. B. St. John Roosa, M. D., LL.D., Professor of Diseases of the Eye and Ear, in the New York Post Graduate Medical School and President of the Faculty; Surgeon to the Manhattan Eye and Ear Hospital; Consulting Surgeon to the Brooklyn Eye and Ear Hospital; formerly Professor of Ophthalmology in the University of the city of New York, and of Diseases of the Eye and Ear in University of Vermont; formerly President of the Medical Society of the State of New York, etc. Sixth edition revised and enlarged. New York, Wm. Wood & Co., 1884.

*A Practical Treatise on Massage;* its history, mode of application, and effects, by Douglas Graham, M. D., Fellow of the Massachusetts Medical Society. Wm. Wood & Co., New York, 1884.

*A Manual of Obstetrics*, by Edward T. Partridge, M. D., Professor of Obstetrics, N. Y. Post Graduate Medical School; Instructor in Obstetrics, College of Physicians and Surgeons; Visiting Physician to the Maternity Hospital, and to the Nursery and Child's Hospital; Gynecologist to the New York Hospital; Fellow of the N. Y. Obstetrical Society. Wm. Wood & Co., New York, 1884.

*A Treatise on the Hemorrhoidal Disease*, giving its history, nature, causes, pathology, diagnosis and treatment, by William Bodenhamer, A. M., M. D. Wm. Wood & Co., New York, 1884.

*A Text Book* of Practical Medicine, designed for the use of students and practitioners of medicine, by Alfred L. Loomis, M. D., LL.D., Professor of Pathology and Practical Medicine in the Medical Department of the University of the city of New York; Visiting Physician to Bellevue Hospital, etc. With ten hundred and eleven illustrations, pp 1102. New York. Wm. Wood & Co., 1884.

*What Shall We Name It?* A Dictionary of Baptismal names for children, By Jno. C. Stockwell, 25 Ann street, New York. Price 25 cents.

*Club-Foot,* Is excision of the Tarsus necessary in Children? By DeForest Willard, M. D., Lecturer Orthopedic Surgery, University of Pennsylvania; Surgeon to the Presbyterian Hospital. Reprint from Transactions of the Medical Society of the State of Pennsylvania, 1884.

*True and False Experts,* By Eugene Grissom, M. D, LL D., Supt. Insane Assylum for North Carolina, Raleigh. Reprint from American Journal of Insanity. July, 1884.

Explanation of the Pathology and Therapeutics of the Diseases of the Nerve Centres, especially Epilepsy. By J. McF. Gaston, M. D., Atlanta, Ga. Reprint from Transactions of the Medical Association of Georgia.

*One Aspect* of the subject of Medical Examination, as set forth in the work of the North Carolina Board of Medical Examiners. Presented by the N. C. Board of Health.

*Reports* of the Trustees and Officers of the Lunatic Asylum of the State of Georgia, from October 1, 1883, to October 1, 1884. J. O. Powell, M. D., Milledgeville, Ga.

*Transactions of the Michigan State Medical Society* for the year 1884. Geo. E. Ranney, M. D., Secretary, Lansing, Mich.

*The Relations of Micro-Organisms to Surgical Lesions.* By Henry O. Marcy, A. M., M. D., Boston. Reprint from Journal Am. Medical Association, Nov. 1, 1884.

*Sixth Annual Report* of the Board of Health of the city of Augusta, Ga. Eugene Foster, M. D., President, Augusta, Ga.

*The Annual Address* before the Literary Societies of Emory and Henry College, Virginia. By Ignatius Shumate, Dalton, Ga.

*The Plaster of Paris Dressing* in the treatment of fractures. By W. O'Daniel, M. D., Bullards, Ga. Reprints from Transactions of Medical Association, Georgia.

*Medical Rhymes.* Selected and compiled from a variety of sources. By Hugo Erichsen, M. D., Professor of Neurology in the Quincy School of Medicine, Medical department of Chaddock College; Licentiate of the Royal College of Kingston, Canada; member of the Detroit Medical Association, corresponding member Natural History Society of Wisconsin, etc. Illustrated. J. H Chambers & Co., St. Louis, Mo., 1884.

*A Text Book* of Pathological Anatomy and Pathogenesis. By Ernst Ziegler, Professor of Pathological Anatomy in the University of Tubingen. Translated and edited for English students. By Donald McAlister, M. D., M. B. Member of Royal College of Physicians, Fellow and Medical Lecturer of St. John's College, Cambridge, Part II. Special Pathological Anatomy, Sections I–VII. Wm. Wood & Co. New York, 1884.

### OUR ADVERTISERS.

*Fredk. Debary & Co., New York.*—These gentlemen are sole agents for the United States for the sale of the celebrated natural mineral waters—Apolinaris and Hunyadi Janos. These waters as sold by this firm are absolutely pure. Refer to their advertisements elsewhere in the Journal, and read the opinions of celebrated authorities.

*Fellows' Syrup Hypophosphites.*—This preparation of hypophosphites has steadily grown in popular favor with the medical profession everywhere. It is largely used in this city, not alone in diseases of the lungs, but as a tonic in debility from whatever cause. See the advertisement and write for sample bottle, use it, and you will be convinced of its merits.

*Goodwyn's Syrup of Hypophosphites.*—This preparation has the endorsement of the best physicians wherever it has been introduced. In our own practice we have been pleased with the results obtained from its use.

*Aloe Hernstein & Co., St. Louis.*—No firm of instrument dealers in the country handle finer instruments than do these gentlemen. We have no hesitancy in indorsing their instruments as being equal in every respect to those of any other manufacture that we know. Any of our readers in need of instruments would do well to write them for catalogue before buying.

*Mellin's Food.*—Dr. Eustace Smith, of London, Physician to the Children's Hospital, and author of "Wasting Diseases of Infants and Children," says: "Mellin's Food is by far the best of any with which I am acquainted. It seems to agree equally well with children whether they are healthy or diseased."

*Jacob's Pharmacy, Atlanta.*—We desire to direct special attention to the attractive advertisement of this house, to be found among our new advertisements in this issue. This is the only house in the South making a specialty of manufacturing fluid extracts from the green roots. Samples of any of these extracts will be furnished physicians on application.

*A. A. Mellier, St. Louis.*—Cort F. Asknew, of Corydon, Indiana, has this to say of the Standard Buggy Case, manufactured by this gentleman: "I am using Mellier's Standard Buggy Case, and consider it the neatest, most durable and most convenient that I have ever seen."

